# Evaluating the impact of a blended interprofessional education course on students’ attitudes towards interprofessional education: a pre-post study

**DOI:** 10.1186/s12909-024-05170-2

**Published:** 2024-02-27

**Authors:** M. Guinat, L. Staffoni, V. Santschi, A. Didier, D. Gachoud, C. Greppin-Bécherraz

**Affiliations:** 1 Department of Intensive Care Medicine, Lausanne University Hospital (CHUV), Lausanne, Switzerland; 2 Medical Education Unit of the School of Medicine FBM, University of Lausanne, Lausanne, Switzerland; 3 Haute Ecole de Santé Vaud (HESAV), School of Health Sciences, HES-SO, University of Applied Sciences and Arts Western Switzerland, Lausanne, Switzerland; 4 La Source, School of Nursing Sciences, HES-SO, University of Applied Sciences and Arts Western Switzerland, Lausanne, Switzerland; 5 Department of Internal Medicine, Lausanne University Hospital (CHUV), Lausanne, Switzerland

**Keywords:** Interprofessional learning, Blended learning, Attitudes, Large-scale interprofessional course, Interprofessional challenges

## Abstract

**Background:**

Since 2011, five educational and healthcare institutions have implemented a short interprofessional education (IPE) course to bring together undergraduates from five disciplines. To meet the logistical challenges of IPE implementation, more specifically, the large number of classrooms needed to gather students together and the need for human resources to guide learning activities, a face-to-face IPE course was redesigned into a blended (online and face-to-face collaborative learning activities) IPE course. In March 2023, 183 medical, 378 nursing, 46 radiologic technology, 69 physiotherapy, and 74 occupational therapy students participated in a one-day IPE blended course to learn interprofessional team functioning and dynamics, role clarification and responsibilities of other professions, and interprofessional communication skills. This study aimed to assess students’ changes in attitudes towards IPE after being involved in a large-scale interprofessional blended learning course.

**Methods:**

A before-after study was conducted using a French translation of the validated questionnaire “University of West of England Interprofessional Questionnaire” (UWE-IP questionnaire). Students’ attitudes towards interprofessional (IP) relationships and IP learning were measured before and after the course. In March 2023, two hundred fifty-six students from five professions answered two subscales of the UWE-IP questionnaire before and after the course (response rate 34%).

**Results:**

Students’ attitudes towards IP relationships improved significantly after the course. The score on this subscale (min 8; max 24) changed from 11.18 (SD 2,67) before the course to 10,38 (SD 2,55) after the course, indicating a significant improvement in attitudes towards IP relationships (*p* < 0,001). More specifically, students had more positive attitudes on the item “I have a good understanding of the roles of different health and social care professionals.” and the item “I feel that I am respected by people from other health and social care disciplines.” after the course. A positive change in students’ attitudes towards IP learning was observed, but the results were not significative.

**Conclusion:**

A face-to-face IPE course redesigned as a blended course helped overcome existing challenges to implementing an IPE course. The results suggest a blended IPE course improves students’ attitudes towards interprofessionality.

## Background

The need for and the value of interprofessional education (IPE) are well recognized in pre- and post-graduate training, improving collaboration and patient care through effective teamwork skills development [1]. Despite strong recommendations to design and implement IPE within healthcare curricula, fostering a pedagogical transition to a sustainable IP culture remains challenging [2, 3]. Educational innovation and the subsequent change in healthcare curricula may be responsible for developing resistance among stakeholders impacted by the change [4]. The high degree of complexity of change within different educational institutions may preclude interprofessional leaders from successfully implementing innovations [5]. Educationalists face numerous challenges while implementing IPE. The literature emphasizes that these challenges should be addressed to successfully implement IPE and enhance students’ engagement in IPE [6]. These challenges include crowded curricula with timetabling restrictions, cost factors with lack of resources [7], lack of accreditation standards [8, 9], resistance to change [4], faculty beliefs that IPE courses need to be added to existing curriculum of each profession rather than integrated and aligned with other professionals outcomes [10, 11]. The strong commitment of the university and leadership of educational institutions with clear policymaker’s support for faculty development positively influence IPE implementation [12–14]. The IPE literature enhances strategic recommendations to approach IPE barriers at the healthcare professions’ micro, meso, and macro levels to accelerate the adoption of IP culture and foster commitment between stakeholders for IPE development [15]. Organizational challenges at the institution’s level are among the first barriers that educators should address [2]. Traditional IPE interventions are delivered face-to-face in sub-group activities through paper-based discussion, problem-based learning [16], and simulation [17]. The exposure to logistical barriers associated with scheduling challenges while planning IP activities across different curricula and geographically distinct sites stressed IPE leaders and designers to innovate in IPE design and implementation [15].

Over the last decade, innovative web-based technologies to deliver IPE have emerged as an adequate strategy to overcome these challenges [18]. In addition, the circumstances surrounding the COVID-19 pandemic underlined the need for increased IPE implementation. They accelerated the transition to online delivery of IPE to balance the social distancing during the pandemic [19]. Online IPE courses seem as effective as face-to-face interaction in acquiring interprofessional knowledge [20]. Previous studies demonstrated that fully online IPE teaching improves students’ attitudes towards interprofessional interaction and interprofessional relationships [21]. Potthoff et al. designed an online introductory IPE course and demonstrated improvement in students’ perceptions of team skills after the course [22].

While the literature provides useful evidence to implement fully online IPE practice to overcome some challenges, some studies mitigate these findings and mention that students may face communication challenges through online learning and perceived busy work and unclear expectations when comparing online courses to traditional in-person courses [20]. As Lestari et al. mentioned, medical, midwifery, and nursing students emphasize that in-person IPE courses may foster stronger cooperation than online learning [23].

These findings support the development of a hybrid method that combines both offline and online IPE teaching approaches. This combined learning strategy may foster the strengths of each approach into a blended strategy. The flipped classroom is a hybrid approach that provides students with online didactic content before-class while using face-to-face in-class sessions to apply their knowledge through different active teaching and learning tasks [24]. This approach increases learners’ motivation and engagement [25], and improves student learning compared to traditional teaching [26]. Implementing the flipped classroom approach in an IPE setting seems promising for developing collaborative practice skills [27, 28]. Raynault et al. pointed out that pre-class online activities should not be restricted to theoretical knowledge teaching [29]. Students should be engaged as soon as possible during pre-class activities through online collaborative tasks that foster IP interaction. In a pre-graduate context, a blended approach, incorporating synchronous and asynchronous technologies in an IPE course, has been developed and seems efficient in improving team-based collaborative skills [30].

While evidence of the effectiveness of blended course design is well recognized in the field of education [25, 31], research on the development of a blended approach in an IPE setting is still limited. Successful IPE implementation aims to improve students’ attitudes towards IPE. We do not know to what extent an IPE blended course influences students’ attitudes towards interprofessionality.

Since 2011, an IPE course delivered in Lausanne, Switzerland, brings together more than seven hundred undergraduate students from five different professions per year. In 2023, to address the logistical challenges of IPE implementation in the Lausanne context, more specifically, the large numbers of classrooms needed to reach students together, timetabling challenges in different academic calendars, misalignment with other IPE course and the need for human resources to guide learning activities, the IPE course was redesigned into a blended IPE course. This actual course comprises online pre-class activities (asynchronous and synchronous online), followed by face-to-face in-class activities.

This study aimed to determine whether a blended interprofessional course improved students’ attitudes towards collaborative learning and interprofessional relationships.

## Method

### Research design

The study was designed as a pre-post study using a French translation of two subscales from the validated questionnaire called the “University West of England Interprofessional Questionnaire” (UWE-IP) to explore changes in students’ attitudes towards IPE before and after the blended course [32, 33].

### Research setting

Since 2011, in Lausanne, Switzerland, a strong partnership between five institutions: the Lausanne University Hospital (CHUV); the Faculty of Biology and Medicine – University of Lausanne (FBM); La Source, School of Nursing sciences, HES-SO University of Applied Sciences and Arts Western Switzerland; the Haute Ecole de Santé Vaud (HESAV), HES-SO University of Applied Sciences and Arts Western Switzerland; and the Faculty of Social Work and Occupational therapy department (HETSL), allowed implementing an IP training course for undergraduate students from five different disciplines.

### IPE blended learning course

The main objectives of this course are to raise healthcare students’ awareness of collaborative practice in initial training, acquire theoretical knowledge on group dynamics and functioning, identify obstacles to effective teamwork, learn how to overcome these obstacles and clarify roles and responsibilities. The IP course was designed following a flipped classroom approach in March 2023 [34, 35]. This blended course consisted of a half day of synchronous and asynchronous online activities and a half day of collaborative learning with face-to-face courses in a sub-group of students. The blended-learning intervention components included:

- Pre-class activities:
  - ◦ Thirty minutes of synchronous presentation of the course using an online connection
  - ◦ Two hours of online course (3 narrative PowerPoint, with e-learning and an online quiz with answers and comments on response available at the end of the asynchronous part)
  - ◦ Two hours of online encounters through online synchronous meetings in the subgroup of IP students without a facilitator to guide them. During this activity, students worked collaboratively to create a poster.
- In-class activities: Five hours: students met face-to-face with the same sub-group they worked with during the online session, guided by a facilitator, to perform exercises that promote clarification of their interprofessional role and cases-based exercises.

The students are guided through the course by a student’s notebook that provides them with the course’s objectives. Technical and IT support created an internet page that centralized online activities, allowing students from different institutions to access the same pre-class content without technical problems. IT support also helped the design group bring students together in online synchronous meetings.

Students were expected to provide a poster as a group production at the end of the online synchronous meeting (pre-class). The latter had to reflect on facilitators and barriers to optimal collaboration embedded in the theoretical knowledge they acquired during the asynchronous part of the course. Then, they presented this poster to their tutors during the face-to-face session. During the face-to-face activities, the tutor asked the IP students’ group to refer to this poster to compare and contrast their healthcare team collaboration to previous reflections. To promote reflection within student groups along the course, students were asked to design a poster was considered a redline that linked pre-class and in-class activities. The aim of the poster was to promote the reflection of IP groups along the course. We hypothesized that this poster helped students self-assess the development of their IP competencies and identify barriers and facilitators to applying these competencies in a healthcare setting. Faculty development was offered to our IP facilitators to help them guide the student’s learning process and enhance reflection within the group [36].

Considering that students of our study have different curricula and academic timetables, efforts were made to align this blended course with pre-existing courses. Some institutions have specific IPE courses that do not involve all the students of the other institutions.

### Participants

This study involved students from five disciplines from different schools and with various levels of training: - medical students from FBM in their first-year Master, - nursing students coming from two different schools: La Source (Nursing school 1) and HESAV (Nursing school 2) in their first year Bachelor, - radiologic technology students from HESAV in their first year Bachelor, - physiotherapy students from HESAV in their first year Bachelor, and - occupational therapy students from HETSL in their first year Bachelor. Finally, all students from HESAV had already participated in an IP course the week before this one. For other students, this IP course was their first exposure to IPE throughout their curriculum. So, nursing students from Nursing School 1 and Nursing School 2 do not have the same exposition to IPE in their own curriculum.

### Instruments

The UWE-IP questionnaire is composed of four subscales. Pollard developed this questionnaire to measure students’ perceptions and attitudes towards interprofessionality [37]. It aims to explore healthcare and social students’ self-evaluation of their communication and teamwork skills and their attitudes towards collaborative learning and collaborative working. In 2016, permission was obtained from the original author, K. Pollard, to translate the questionnaire into French. Researchers (with healthcare background) coming from the four institutions involved in the design of the course translated in French two of the 4 UWE IPQ subscales: 1) the Interprofessional Learning Scale, which investigates students’ attitudes towards IP learning, 2) the Interprofessional Relationship Scale, which evaluates students’ perceptions of interactions between different health and social care professionals. These two subscales of this questionnaire were selected because they related most directly to the outcomes of the course. The translation and cultural adaptation of the UWE IQ instrument were carried out according to the methodology proposed by the World Health Organization (WHO 2016): translation original version/French, review of the translation by a group of experts, translation French/original version, pre-testing and a cognitive debriefing with a sample of students from the six professions [38]. Indeed, after translation from English to French, this French version was submitted to four experts in IPE to reach a consensus for each item in the Swiss-French context. Then, the two sub-scales were translated back into English by a bilingual healthcare worker with English as their mother tongue, who had not seen the other version of the subscale. This English version was compared to the original version of the two sub-scales by the members of the implementation team of the scale to ensure that the underlying concepts were similar between both versions. Then, the French version of the two sub-scales was submitted to 26 students from 6 different professions for a cognitive interview to verify the understanding of the items and refine wording. Minor modifications were made to the translation. The final version of the two translated subscales was then submitted to 12 experts for an item evaluation (the content validity index of the two sub-scales was 0.86). Finally, psychometric tests were conducted on the two subscales completed by 361 of the 530 students involved in the interprofessional training program in order to validate the translated subscales.

The Interprofessional Learning and Relationship Scales are composed of 9 and 8 items, respectively (Table 1). Statements are scored using a five-point Likert scale from 1 (strongly agree) to 5 (strongly disagree), including the neutral point. The Interprofessional Learning Scale has a minimum score of 9 (most positive attitudes) and a maximum score of 45 (most negative attitudes). The Interprofessional Relationship Scale has a minimum score of 8 (most positive attitudes) and a maximum score of 40 (most negative attitudes). The lower the score is, the more students have a positive attitude towards interprofessional education.

**Table 1:** The two sub-scales of the UWE-IP questionnaire (French translation)

Subscales of UWE-IP	Involved items
**Interprofessional Learning Scale**	**This subscale investigates students’ attitudes towards IP learning**
1. My communication skills with patients/clients would be improved through learning with students from other health and social care professions. *(Mes compétences de communication avec les patients/clients s’amélioreraient grâce à des apprentissages avec des étudiants d’autres professions de la santé et du social)* 2. My communication skills with other health and social care professionals would be improved through learning with students from other health and social care professions. *(Mes compétences de communication avec les professionnels de la santé et du social s’amélioreraient grâce à des apprentissages avec des étudiants d’autres professions de la santé et du social)* 3. I would prefer to learn only with peers from my own profession. (R) *(Je préfèrerais apprendre uniquement avec les pairs de ma propre profession)* 4. Learning with students from other health and social care professions is likely to facilitate subsequent working professional relationships. *(Apprendre avec des étudiants d’autres professions de la santé et du social facilite probablement les futures relations professionnelles)* 5. Learning with students from other health and social care professions would be more beneficial to improving my teamwork skills than learning only with my peers. *(Apprendre avec des étudiants d’autres professions de la santé et du social serait plus bénéfique pour améliorer mes compétences de travail en équipe plutôt qu’apprendre uniquement avec les pairs de ma propre profession)* 6. Collaborative learning would be a positive learning experience for all health and social care students. *(L’apprentissage collaboratif serait une expérience positive d’apprentissage pour tous les étudiants de la santé et du social)* 7. Learning with students from other health and social care professions is likely to help to overcome stereotypes that are held about the different professions. *(Apprendre avec des étudiants d’autres professions de la santé et du social aide probablement à surmonter les stéréotypes à l’égard des différentes professions)* 8. I would enjoy the opportunity to learn with students from other health and social care professions. *(J’apprécierais l’opportunité d’apprendre avec des étudiants d’autres professions de la santé et du social)* 9. Learning with students from other health and social care professions is likely to improve the service for patient/client. *(Apprendre avec des étudiants d’autres professions de la santé et du social améliore probablement les prestations au patient/client)*	
**Interprofessional Relationship Scale**	**This subscale evaluates students’ perceptions of their relationships with other health and social care professionals**
10. I have an equal relationship with peers from my own professional discipline. *(J’ai une relation d’égal à égal avec les pairs de ma propre profession)* 11. I am confident in my relationships with my peers from my own professional discipline. *(J’ai confiance dans mes relations avec les pairs de ma propre profession)* 12. I have a good understanding of the roles of different health and social care professionals. *(J’ai une bonne compréhension des rôles des différents professionnels de la santé et du social)* 13. I am confident in my relationships with people from other health and social care disciplines. *(J’ai confiance dans mes relations avec des personnes d’autres professions de la santé et du social)* 14. I am comfortable working with people from other health and social care disciplines. *(Je suis à l’aise de travailler avec des personnes d’autres professions de la santé et du social)* 15. I feel that I am respected by people from other health and social care disciplines. (*Je sens que je suis respecté.e par les personnes d’autres professions de la santé et du social)* 16. I lack confidence when I work with people from other health and social care disciplines. (R) *(Je manque de confiance quand je travaille avec des personnes d’autres professions de la santé et du social)* 17. I am comfortable working with people from my own professional discipline. (*Je suis à l’aise de travailler avec des personnes de ma propre profession)*	

(R) Reverse coded item

### Data collection and analysis

Quantitative data were collected from undergraduate students two weeks before the course (pre-test) and two weeks after the course (post-test) using an online questionnaire. All students received a reminder e-mail to answer the questionnaire one week after the first mailing. Demographic data such as gender and age were collected. A simplified Likert scale from 1 to 3 points was used for statistical analysis. The analysis was performed using the sum of the scores obtained in each of the two subscales of the UWE-IP questionnaire. The analysis was performed for each subscale, then for each item of the subscales, and finally, the analysis was made based on the students’ professions. A decrease in the mean score from the pre-test to the post-test indicates an improvement in the measure, which means a more positive attitude after the course. A student t-test was used to compare the UWE-IP score between the pre-test and post-test for each subscale and the total of the two subscales. A Wilcoxon signed-rank test was used to compare the pre-test and post-test scores of each subscale item. A paired-sample t-test was used to compare the scores obtained by students based on their profession between the pre-test and post-test. A Wilcoxon signed-rank test was used to calculate the difference if the sample size was less than 30 in the student profession. All reverse-coded items were recoded and then analyzed. Data were analyzed using SPSS version 23. A *p*-value of α ≤ 0.05 was considered statistically significant.

### Ethical considerations

The Cantonal Ethics Committee Vaud waived the need for ethics approval (BASEC-No Req-2023–00575).

## Results

### Participants’ demographics and response rate

The study was conducted in March 2023. Table 2 shows the participant’s demographic characteristics. The response rate was 34%. The majority of students were female and between 21 and 25 years old.

**Table 2:** Demographic table and response rate

Number of students involved in the course		750
Number of students who answered the questionnaire (% of total number of students)		256 (34%)
Gender		
Female		198
Male		49
No response		9
Age		
18–20 years		47
21–25 years		149
26–30 years		30
31–35 years		7
> 35 years		20
No response		3

Table 3 provides information on the number of students from each profession involved in the IP course per year. It summarizes the response rate of each discipline to the UWE-IP questionnaire.

**Table 3:** Percentage of students per discipline participating in the course and response rate to the questionnaire

Discipline	Students involved in the IPE course	Number of responders (% response rate per discipline)
Medicine	183	75 (41%)
Nursing	378	128 (39%)
Physiotherapy	69	16 (23%)
Radiologic technology	46	10 (22%)
Occupational therapy	74	27 (36%)
Total student number	750	256

Nursing and medical students are widely represented in our sample, and the number of students in these professions exceeds all other professions in each subgroup of students. This distribution is explained by the number of students enrolled in each school for undergraduate training. Radiologic technology students are the least represented.

### Students’ attitudes towards interprofessional education using pre-post intervention method

Table 4 shows the results of the pre-test and post-test questionnaires completed by students before and after the IP course. A t-test indicated that the Mean (*M)* of total scores on the two subscales decreased significantly between the pre-test and the post-test (*M* = 22.26 to *M* = 21.41; *p* = .001). These results showed an increase in positive attitudes towards IP education after the IPE course.

**Table 4:** Differences between the pre-test and post-test of UWE-IP subscales scores for all students

Subscales		Pre-test Mean M (SD)	Post-test Mean M (SD)	t-test (df), *P*-value
Interprofessional learning scale (score min:9; max:27)				
2023		11.08 (2.73)	11,03 (2.9)	0.289 (255), 0.77
Interprofessional relationship scale (score min: 8; max:24)				
2023		11.18 (2,67)	10.38 (2.55)	5.3 (255), 0.001
Total of the two subscales (score min: 17; max: 51)				
2023		22.26 (3.96)	21.41 (4.30)	3.311 (255), 0.001

*M*  Mean, *SD* Standard deviation

### Change in students’ attitudes towards interprofessional learning and interprofessional relationship using pre-post intervention method

The two subscales were analyzed separately (Table 4). For the IP learning subscale, changes in attitudes towards IP learning were not statistically significant (*M* = 11.08 to *M* = 11.03; *p* = .77).

However, the score analysis for the relationship subscale demonstrated a significant decrease in the mean score between the pre-test and the post-test (*M* = 11.18 to *M* = 10.38; *p* = .001). Thus, students’ attitudes towards interprofessional relationships increased significantly after the course.

### Change in students’ attitudes towards interprofessional learning and interprofessional relationships based on each item of the scale using pre-post intervention method

Table 5 presents the items of each subscale, for which there was a significant statistical improvement in scores between the pre-test and post-test. Students had more positive attitudes towards item 12, “I have a good understanding of the roles of different health and social care professionals.” And item 15, “I feel that I am respected by people from other health and social care disciplines.” After the course. This table shows that items with a statistical improvement are classified in the interprofessional relationship scale.

**Table 5:** Item of each of the two subscales with a significant decrease in mean score from pre-test to post-test

Interprofessional learning scale Z; *p*-value		
Item 1		− 0.804; 0.42
Item 2		− 0.452; 0.651
Item 3		-2.246; 0.025
Item 4		-1.156; 0.248
Item 5		− 0.189; 0.850
Item 6		− 0.878; 0.380
Item 7		− 0.324; 0.746
Item 8		− 0.865; 0.387
Item 9		-1.844; 0.065
Interprofessional relationship scale Z; *p*-value		
Item 10		-1.477; 0.14
Item 11		− 0.477; 0.633
Item 12		-5.605; <0.001
Item 13		-1.712; 0.087
Item 14		-2.496; 0.013
Item 15		-3.335; <0.001
Item 16		-1.872; 0.061
Item 17		-1.975; 0.048

A *P*-value of α ≤ 0.01 is considered statistically significant

### Change in students’ attitudes by discipline and institution using pre-post intervention method

Table 6 shows the results of the change in attitude towards interprofessional education before and after the blended IPE course for each profession. Results for radiologic technology students are not available separately for the two subscales due to the low number of students.

**Table 6:** *Differences between the pre-test and post-test of UWE-IP two subscales scores per discipline*

Total score from two Subscales of UWE-IP	Pre-test	Post-test	Test (df), *p*-value
Nursing school 2 Mean (Standard Deviation)			
Interprofessional Learning Scale	11.27 (2.41)	11.67 (3.29)	t -0.688 (32), 0.496
Interprofessional Relationship Scale	10.52 (2.85)	9.94 (2.5)	t 1.13 (32), 0.265
Total score from two Subscales of UWE-IP	21.79 (4.05)	21.61 (5.03)	t 0.182 (32), 0.86
Nursing school 1 Mean (Standard Deviation)			
Interprofessional Learning Scale	11.48 (3.13)	11.23 (2.95)	t 0.94 (94), 0.351
Interprofessional Relationship Scale	10.94 (2.52)	10.54 (2.37)	t 2.001 (94), 0.048
Total score from two Subscales of UWE-IP	22.42 (4.04)	21.77 (4.08)	t 1.82 (94), 0.07
Physiotherapy Mean (Standard Deviation)			
Interprofessional Learning Scale	10.69 (1.3)	10.63 (1.9)	Z -0.443, 0.658
Interprofessional Relationship Scale	11.5 (2.97)	9.06 (1.24)	Z 2.95, 0.003
Total score from two Subscales of UWE-IP	22.19 (3.45)	19.69 (2.55)	Z -2.88, 0.004
Medicine Mean (Standard Deviation)			
Interprofessional Learning Scale	10.72 (2.76)	10.67 (3.04)	t 0.145 (74), 0.885
Interprofessional Relationship Scale	11.52 (2.75)	10.84 (3.15)	t 2.331 (74), 0.022
Total score from two Subscales of UWE-IP	22.24 (4.23)	21.51 (4.85)	t 1.42 (74), 0.16
Radiologic technology Mean (Standard Deviation)			
Interprofessional Learning Scale	Not available		
Interprofessional Relationship Scale	Not available		
Total score from two Subscales of UWE-IP	24 (4.42)	22.6 (4.97)	Z -1.016, 0.31
Occupational therapy Mean (Standard Deviation)			
Interprofessional Learning Scale	10.22 (1.48)	10.44 (2.08)	Z -0.42, 0.674
Interprofessional Relationship Scale	11.52 (2.53)	9.78 (1.45)	Z -3.34, < 0.001
Total score from two Subscales of UWE-IP	21.74 (2.88)	20.22 (2.46)	Z -2.81, 0.005

## Discussion

The literature provides strong recommendations for implementing IPE in each curriculum. Still, designers and educational leaders should anticipate the challenges they may face while designing such IPE courses. Blended learning strategies offer an opportunity to manage these challenges. There is growing evidence that blended learning can be effectively implemented in an undergraduate healthcare setting [39], positively impacting students’ collaborative practice [29, 40].

This study aimed to determine whether participation in a one-day large-scale IPE blended course improved students’ attitudes towards interprofessional education. For this purpose, in this study, a pre-post intervention quantitative research design was adopted. Our results suggest that participation in a blended IP course delivered to more than 700 students enhances students’ attitudes towards interprofessionality with a more positive attitude after the course. This is aligned with previous literature that mentions that students’ attitudes towards interprofessionality improve after a blended course with effective teaching of interprofessional team process skills (knowledge, skills, and attitudes) and Carbonaro et al. demonstrated that a blended course seems to be as effective as a IP face-to-face-course to achieve IP learning outcomes [30].

The results were not significant for the IP learning scale through the analysis by sub-scale of the UWE-IP questionnaire. On the contrary, a significant improvement in students‘ attitudes towards IP relationships was observed. Although the results are statistically significant, we observed a small amplitude of the change in scores before and after the course. This may be explained by the fact that students start the course with a positive attitude, which leaves little room for improvement.

However, these findings corroborate the results of Evans et al., who demonstrated that a fully online IPE course delivered to six different undergraduate professions had significant positive changes for interprofessional relationships but no significant changes for IP learning [21].

To better understand the results of this study and analysis by sub-scales, it is important to notice that the main items of the IP relationship scale of the UWE-IP questionnaire investigate students’ relationships with peers and understanding of other professions’ roles. In that sense, our results align with another study showing that students better understand each profession’s role and how these roles complement each other after enrolling in an online IP case-based learning course [23]. In our study, students demonstrated more positive attitudes on item 15, related to understanding the roles of different health and social care professionals after the IPE course. Other studies showed that online IPE courses contributed to achieving learning outcomes related to professions’ role understanding [22, 30]. Relationship scale score in our study might have been positively influenced by the outcomes of the online activities during which students must collaboratively work, such as creating the poster. Aside from this, the effective design of online IPE courses requires active engagement in online activities [29]. In our study, the poster aimed to help students connect the theoretical content learned during the online session with the practical examples they encountered during the face-to-face session. In addition, during the poster collaboration, students are engaged in group reflection on collaborative teamwork relationships. Breaking down barriers is facilitated by active and deep students’ reflection on their own beliefs [41]. Reflection on the IPE process facilitates reaching IP educational outcomes [36] and facilitates optimal integration of interprofessional perspectives within and alongside the uniprofessional development. This socialization process based on reflection in practice might create an environment conducive to trusting interprofessional relationships [10]. We make the hypothesis that integrating this poster, promoting interprofessional reflection within a sub-group of students, may have positively influenced students’ relationships after the course by fostering interactions between students and active engagement in the learning task.

Unlike the relationship scale, a lack of significant improvement in the IP learning scale scores was noticed in this study, and previous literature and characteristics of the scale may explain these results. Indeed, the main items of the IP learning scale of the UWE-IP questionnaire investigate communication and teamwork skills. Some studies highlight that students deplore the lack of interaction between students using online courses [22]. In our study, even if students met together in online group sessions, this online component of the course may challenge students’ communication skills development and preclude optimal student interactions. Jones et al. demonstrated that, after converting an existing IPE course to an online learning environment during the COVID-19 pandemic, students valued online courses as effectively as face-to-face courses [20]. However, according to the qualitative results of focus groups, the students deplored the lack of engagement and also expressed that communication was challenging in online sessions. On the other hand, literature underlined that during an online IP course, students could practice IP collaboration and communication through online discussion [23]. The different design models of the online course may explain these different findings and need further attention.

In our study, the absence of a facilitator to guide sub-groups of students during the online synchronous meeting might be another factor that hinders significant improvement in students’ attitudes towards IP learning. Designers in online IPE should stimulate students to think, plan, do, and reflect within IPE intervention to acquire appropriate skills [42]. As teachers in IPE, facilitators play key roles in this reflective practice, fostering collaborative development [43]. The literature demonstrates a growth in developing online IPE facilitation to support students’ reflection in these online activities with specific faculty development to empower the change [44, 45]. Conversely, facilitation in the online environment might provide some technical challenges. Still, the involvement of a facilitator seems to contribute to more positive experiences for the students and enhances their participation. Finally, constructive alignment [46] appeared to be a relevant approach to examine the items of the two sub-scales that did not show improvement after the course. As a reminder, our research team specifically chose these two UWE questionnaire subscales because they align well with the course’s objectives. As we followed constructive alignment principles while designing the course, the lack of improvement in certain items may indicate that some learning objectives have not been achieved. We could hypothesize that our blended course could not cover each course outcome equally and that some objectives were less emphasized. In addition, our blended course provides students with only a short exposure to teaching IP learning, a limited exposure that might have hindered improvements in the score for these specific items.

However, despite the lack of improvement in attitudes towards IP learning, we observed that students start the course with strong positive attitudes. This may be explained by the fact that the youngest students are enrolled in the course, and early involvement may prevent them from developing stereotypes of other professions [47]. Our results align with previous literature that found that students’ readiness for interprofessional learning was high at the beginning of their curriculum before being involved in an IPE course [48]. In addition, this corroborates a study that suggests that developing an interprofessional identity in parallel to a professional identity is not exclusive [10]. According to the interprofessional socialization framework [49], exposition to the IPE course contributes to breaking down the professional silos and stereotypical perceptions. Even if students’ attitudes are positive at the beginning of the course, some authors argue that IPE should be introduced early in the curriculum both to capitalize on positive student attitudes and to avoid the decline of attitudes due to socialization within one’s own profession [48]. The lack of observed effect on attitudes towards learning may be influenced by prior experience of the students. Students’ background and prior exposure to IPE are factors that influence students’ attitudes toward IPE [50]. However, our current data and analysis do not allow us to analyze the specific factors affecting students’ attitudes toward IPE.

Students’ attitudes towards interprofessionality evolved differently depending on their professions. Indeed, physiotherapy and occupational therapy students showed a significant trend in positive attitude after the course. We noticed a more positive attitude after the course for other professions, but the results were not significant. Through the analysis of the two sub-scales, a significant improvement in students‘ attitudes towards IP relationships was observed, except for nursing students coming from the Nursing school 2. The results of the professional analysis were not significant for the IP learning scale.

In traditional IPE courses, students’ backgrounds influence their attitudes towards IP learning [50, 51]. However, only a few studies showed various changes in attitudes depending on the students’ professions after an IPE online course. Lestari et al. demonstrated, through a mixed method, that nursing students’ scores for constructive, collaborative, and motivational activities evaluations were the lowest compared to other professions after an online IP case-based course. The qualitative results emphasized that nursing students lack the skills to lead discussions through online sub-group activities due to their traditional lecture-based curriculum [23].

The development of a IP blended course seems to effectively improve attitudes towards IP relationship for students who participated to the blended course. Blended learning becomes a learning strategy that educators in IPE should investigate to teach interprofessional competencies. Further studies are necessary to understand better which characteristics of the course and instructional design factor of a blended learning course might contribute to student attitude improving. Other studies are also needed to explore students’ perspectives on blended learning in an IPE context. For example, qualitative research could help understand how blended learning approaches help students improve their attitudes towards IPE relationships and what prevents them from significantly improving their attitudes towards IP learning.

### Limitations and strengths

Our study presents several limitations. There is no control group for comparison. For this reason, we cannot claim that the blended course was the only condition that allowed the students’ attitudes to become positive. Even if the sample size was large, students came from only one pregraduate healthcare context with a specific course design, so the results might not be directly applied to other contexts and other IPE blended courses. The authors were actively engaged in the course’s design and teaching. As authors of this article and students involved in the blended course share the same learning environment, this might lead to power issues and social desirability bias. To address these biases, data were anonymized and collected through online questionnaires sent by an independent institution, distinct from the institutional affiliation of the students involved in the course. Another limitation inherent in our study exists while examining the response rate of the questionnaire and investigating the characteristics and motivation of the students who answered the questionnaire. The students who answered the questionnaire may represent both highly motivated students who appreciated the course and those who less enjoyed it [52]. It is challenging to encompass this fact in interpreting the results of this study.

Our study contributes to the understanding of blended courses’ effectiveness in IPE. This is crucial since such educational approaches are being developed in education, specifically in the IPE setting. Our study offers an example of implementing a large-scale course involving five different professions. The substantial number of students enrolled in the course and the response rate to the questionnaire provided us with a good representation of the participants and improved the generalizability of the results. This study emphasizes an emerging area of research focusing on the effectiveness of innovative design in IPE.

## Conclusion

Using digital technologies to reach students from different disciplines together offers opportunities to solve challenges in implementing IPE courses, specifically logistical barriers and timetables scheduling. A blended IPE course (online and face-to-face) is an effective learning and collaborative method to teach IP competencies to students from five different fields with the improvement of their attitudes towards IP relationships. Further research should be designed to compare the blended learning strategy to traditional IPE courses. Qualitative research should help educators understand which design factors of the IPE blended course contribute to improving students’ attitudes towards IPE after being actively involved in the course.

## Data Availability

No datasets were generated or analysed during the current study.

## Abbreviations

IP: Interprofessional
IPE: Interprofesisonal Education
UWE-IP: University West of England Interprofessional Questionnaire
*M*: Mean
